# Prediction of Acute Liver Injury Trajectory in Patients Following Acetaminophen Overdose: A Multibiomarker Machine Learning Proof‐of‐Concept Study

**DOI:** 10.1002/cpt.70320

**Published:** 2026-05-11

**Authors:** Chris Humphries, Alastair M. Kilpatrick, Kathleen M. Scullion, Rhona Aird, Lorraine Bruce, Maria Elena Candela, Tak Yung Man, Stuart J. Forbes, James W. Dear

**Affiliations:** ^1^ The University of Edinburgh Centre for Cardiovascular Science Edinburgh UK; ^2^ The University of Edinburgh Centre for Precision Cell Therapy for the Liver Edinburgh UK; ^3^ The University of Edinburgh Centre for Regenerative Medicine Edinburgh UK

## Abstract

Clinical translation of novel therapies can be hindered by heterogeneity‐driven sample size inflation in late‐stage trials. In acetaminophen‐induced liver injury (APAP DILI), many patients recover spontaneously, diluting investigational drug efficacy signals. We developed a prognostic enrichment tool to identify patients with worsening injury trajectories for more efficient trial designs. Biomarker model discovery and evaluation used serum samples from three UK cohorts: the MAPP2 APAP DILI biobank (*n* = 147), an independent pre‐intervention evaluation cohort from the ongoing MAIL trial (*n* = 34), and healthy controls (*n* = 13). We measured 63 biomarkers and evaluated 321,682 combinations using kernel naïve Bayes classification to predict liver injury trajectory (ALT rising vs. falling). Sensitivity analysis using patient‐level grouped cross‐validation showed combining multiple biomarkers while constraining collinearity was necessary to maximize performance. A four‐biomarker model (MCSFR, WBC, Sodium, K18) achieved AUC 0.868 (derivation) and 0.854 (evaluation). When optimized for prognostic certainty, the model yielded a Positive Likelihood Ratio of 14.4, increasing the Positive Predictive Value for worsening injury from a baseline of 29.4% to 85.7%. Time‐dependent cost‐minimization modeling for a hypothetical phase 3 trial identified an application threshold (sensitivity 80.0%, specificity 91.7%, Number Needed to Screen 3.4) as the global economic optimum, resulting in an illustrative trial cost reduction from $39.0 M to $8.3 M. This proof‐of‐concept demonstrates multidimensional biomarker models can resolve signal dilution. Distinguishing patients destined for injury progression reduces sample size requirements, which could de‐risk novel therapy development.

**Study Highlights**

**WHAT IS THE CURRENT KNOWLEDGE ON THIS TOPIC?**

The development of new therapies for acute organ injury (such as liver toxicity) can face prohibitive costs associated with high sample size requirements. These large sample sizes are necessitated by the heterogeneity of the patient population, where spontaneous recovery is common.

**WHAT QUESTION DID THIS STUDY ADDRESS?**

We sought to identify whether the trajectory of liver injury could be predicted to a degree which could result in significant reduction in clinical efficacy trial costs.

**WHAT DOES THIS STUDY ADD TO OUR KNOWLEDGE?**

We demonstrated that a four‐biomarker candidate signature (MCSFR, WBC, Sodium, K18) can predict injury trajectory (AUC 0.854) and could effectively triple the concentration of patients with worsening injury in a future trial population (Enrichment Ratio 2.9×).

**HOW MIGHT THIS CHANGE CLINICAL PHARMACOLOGY OR TRANSLATIONAL SCIENCE?**

Implementing this modeling approach as a screening tool could allow trialists to exclude patients destined for spontaneous recovery. This enrichment strategy could mitigate signal dilution from patients destined to spontaneously recover and reduce costs significantly, de‐risking the commercial development of novel therapies.

Drug‐induced liver injury (DILI) remains a leading cause of acute liver failure (ALF) in high‐income countries, with acetaminophen (APAP) overdose accounting for the majority of cases.^1^, ^2^ While N‐acetylcysteine (NAC) is an effective antidote early in the course of poisoning, it has limited efficacy once hepatocellular necrosis is established.^3^, ^4^, ^5^ Consequently, research has pivoted toward novel therapeutics targeting downstream injury pathways, including secondary mediators of injury such as fomepizole, and immune modulation and regenerative cell therapies such as alternatively activated macrophages (AAMs).^6^, ^7^, ^8^, ^9^, ^10^

However, a critical bottleneck in the translation of these novel therapies is the prohibitive cost and risk associated with Phase 2 and 3 clinical trials. In acute liver injury, patient outcomes are highly heterogeneous; a significant proportion of patients will recover spontaneously with standard supportive care, yet the fragmented nature of existing clinical and public health datasets limit identification of reliable prognostic indicators from routine data alone.^4^, ^5^, ^11^ This high rate of spontaneous resolution creates substantial signal dilution. If a drug is effective, but it is tested in a population where 70% of patients improve regardless, the observed treatment effect is mathematically diluted toward zero.

This challenge is exemplified by the ongoing MAIL (Macrophages for Acute Liver Injury) trial, a Phase 1 study evaluating allogeneic macrophage therapy for APAP DILI.^8^ While this safety and tolerability trial targets patients with severe injury (ALT >1,000 IU/L), the variable natural history of DILI means that a substantial fraction of recruits may already be in the early phase of recovery at the time of enrolment. In a future efficacy study, this spontaneous resolution would dilute the treatment signal, necessitating a large sample size to prove benefit. Similar issues have been faced for the translation of calmangafodipir to clinic following a successful Phase 1 trial.^12^

To overcome this translational “valley of death,” there is a need for prognostic enrichment tools capable of identifying, at a single timepoint, the specific subset of patients destined for injury progression. Such tools would allow trials to selectively recruit those most likely to benefit, thereby reducing sample size requirements and de‐risking the development of novel interventions.

Individual biomarkers have struggled to accurately stratify the acute liver injury patient population.^13^ In this study, we aimed to develop a multidimensional biomarker model to serve as a prototype for clinical trial enrichment. We applied a “mechanistic triangulation” approach to predict injury trajectory and performed a statistical power simulation to quantify the specific impact of this tool on limiting signal dilution and reducing clinical trial costs.

## MATERIALS AND METHODS

### Design and setting

**Figure 1:**
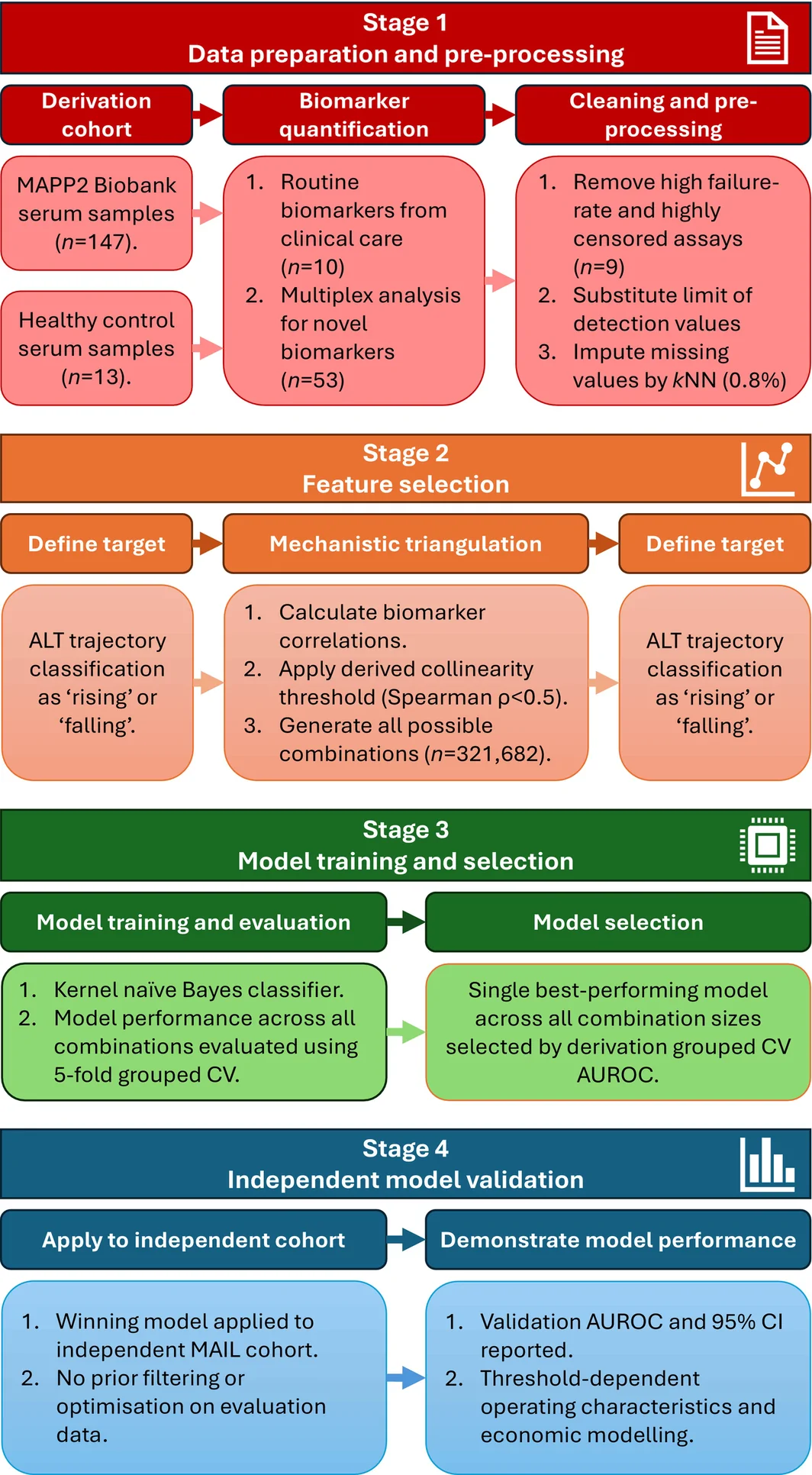
Study flow diagram. ALT, Alanine Aminotransferase; AUROC, Area under the receiver operating characteristic curve; CV, Cross‐validation; *k*NN, *k‐*nearest neighbor imputation.

This was a biomarker discovery and evaluation study conducted using human serum samples from three distinct UK‐based cohorts (**Figure** **1**). Discovery utilized samples from the multicenter MAPP2 biobank, with a cohort of healthy donor samples used to standardize biomarker values. Model performance was evaluated on an independent cohort of pre‐intervention samples collected pre‐intervention from participants in the ongoing Macrophages for Acute Liver Injury (MAIL) trial; ISRCTN12637839.^8^ All patients received routine clinical care (**Figure** **1**).

### Study cohorts and sample processing

Discovery samples from MAPP2 biobank APAP DILI participants (July 2018–May 2023; no decedents in biobank) were selected by peak alanine aminotransferase (ALT): no ALT rise (peak <50 U/L), sub‐hepatotoxic DILI (50–999 U/L), and hepatotoxic DILI (≥1,000 U/L). All samples from a patient were assigned to that patient's phenotype regardless of timepoint ALT. Thirteen healthy donor samples established reference ranges via median‐adjusted deviation (MAD) Z‐scores. Independent evaluation samples from MAIL trial (October 2023–April 2025) were obtained post‐hepatotoxicity development, and prior to any trial interventions.

MAPP2 (hepatotoxicity derivation cohort; 73 samples, 2–8 samples per patient) overdose presentations comprised 75% acute ingestions (15/20 patients, of which 5 were ≤8 hours from ingestion to NAC, and 10 were >8 hours from ingestion to NAC), 15% supra‐therapeutic overdoses, and 10% staggered intentional overdoses. MAIL (evaluation; 34 samples) overdose presentations comprised 60% acute ingestions (6/10 patients, all >8 hours from ingestion to NAC), 30% supra‐therapeutic overdoses, and 10% staggered intentional overdoses.

Patients with less severe DILI are discharged earlier, resulting in baseline temporal differences between cohorts. This was addressed by grouping samples by patient phenotype for some analyses while examining temporal profiles in others. As an exploratory biomarker discovery study, a sample size calculation was not performed, no protocol was prepared, and study‐specific patient and public involvement was not undertaken. Evaluation cohort size was determined by the number of eligible participants with appropriate samples available from the MAIL trial.

To prevent within‐patient data leakage from serial samples, all cross‐validation during model discovery used patient‐level grouped fivefold cross‐validation (caret::groupKFold). Fold assignments were computed once with a fixed random seed prior to model search, ensuring all model combinations were evaluated on identical data splits.

### Biomarker quantification

Biomarkers were selected through literature review, local consensus, platform availability, and serum volumes. All 63 biomarkers and assays are listed in **Supporting Information** **S1**. To ensure cross‐platform validity, we assessed the concordance of CCL2 quantification between Luminex and Mesoscale Discovery assays, observing a strong correlation (ρ = 0.881) (**Supporting Information** **S2**).

Discovery samples were analyzed in singlicate with duplicate standards, consistent with European Bioanalysis Forum recommendations.^14^ Evaluation samples were analyzed in duplicate. All assays were undertaken by technicians blinded to sample phenotypes through the use of pseudonymization codes. Sample aliquoting and de‐pseudonymization was undertaken by a non‐blinded study team member.

### Statistical and bioinformatic analysis

#### Data pre‐processing and management

Where volume permitted, analyses used *n* = 160 samples, except Luminex cytokines (*n* = 159), HMGB1 ELISA (*n* = 159), and miR‐122 RT‐PCR (*n* = 159). Unreadable values from analyzer failure were removed (*n* = 1 for CCL5, MCSFR, PDGF‐BB, impacting a single sample). CX3CL1 and CXCL14 were excluded due to all measurements (including healthy controls) falling on standard curve lower plateaus, indicating that the assay platform lacked the sensitivity to detect physiological levels in this context.

Biomarkers with ≥50% censored data (outside quantification limits) were excluded due to insufficient quantitative resolution (CCL8, IL‐1a, IL‐1b, IL‐3, IL‐4, IL‐21, IL‐23). For remaining biomarkers (*n* = 44), censored data were substituted with detection limits plus small constants (±0.1) to maintain rank values. Missing values were imputed using kNN (VIM package, *k* = 5). For final model development, imputation was repeated after restricting to 15 biomarkers available in both cohorts. The discovery matrix required imputation for 0.8% of data points; the evaluation matrix required none.

While the discovery cohort included samples across the full injury time‐course, the evaluation cohort comprised exclusively pre‐intervention samples from patients meeting trial eligibility criteria. The evaluation cohort therefore represents an actual decision‐point population encountered by a trialist. To ensure the derived model could be evaluated on an external cohort, we applied a strict translational filter prior to model assembly. Imputation was repeated after restricting the dataset to the 15 biomarkers explicitly available in both the discovery (MAPP2) and independent evaluation (MAIL) cohorts. The discovery matrix required imputation for 0.8% of data points; the evaluation matrix required none.

#### Modeling and statistics

Analyses used R (v4.4.2). Group comparisons used Kruskal–Wallis with *post hoc* Dunn's tests and Bonferroni correction (*P* < 0.05). Temporal relationships between biomarkers and ALT decline assessed post‐peak samples, categorizing biomarker decline as “Earlier,” “Later,” or “Same” relative to ALT.

For dimensionality reduction, data were scaled and centered. Spearman correlation matrices were computed and visualized (corrplot v0.95). Principal Component Analysis used eigendecomposition; UMAP used uwot package (v0.2.3, n_neighbours = 15, min_dist = 0.2).

Supervised models used kernel naïve Bayes (e1071 v1.7–14) to classify subsequent ALT trajectory (rising vs. falling). To select uncorrelated features, sensitivity analysis identified optimal Spearman correlation thresholds. While the initial 63‐biomarker panel offered a vast search space (>300,000 potential models), we focused our exhaustive search on the translationally validated subset. We evaluated all biomarker combinations (size 1–8) within this restricted panel that met collinearity thresholds (*n* = 4,159 unique models). Performance was assessed using mean patient‐level grouped CV AUC as primary metric (pROC v1.19.0.1). The single top‐performing model at derivation was then applied to the independent evaluation cohort for the purposes of economic evaluation. Evaluation AUC 95% CIs were calculated using 2000 bootstrap replicates.

We employed a kernel Naïve Bayes classifier, selected *a priori* for its robust performance in high dimension, low sample size settings and its compatibility with the feature independence constraint imposed by our correlation threshold optimization. Our feature selection strategy (correlation filtering, exhaustive search) was specifically designed around this classifier's assumptions. Consequently, direct comparison with alternative classifiers would require complete re‐optimization of feature selection criteria and would not represent a fair methodological comparison. Unlike Gaussian Naïve Bayes, which assumes data follow a normal distribution, the kernel approach accommodates the non‐parametric, often skewed distributions frequently observed in biological data, thereby preventing model bias driven by outliers or non‐linear physiologic responses.

#### Economic optimization and feasibility modeling

To determine the optimal decision threshold for trial enrichment, we developed a time‐dependent economic cost function rather than relying solely on standard diagnostic metrics. We modeled the total trial cost as a function of screening sensitivity, integrating three distinct cost drivers: screening costs ($500 per patient), randomization/treatment costs ($30,000 per patient), and fixed operational costs ($3 million per year). The trial duration was dynamically calculated based on a global recruitment capacity of 350 screens per year. This approach credibly penalizes strategies that require excessive screening (high specificity), as the resulting extension in trial duration incurs prohibitive fixed operational costs. We simulated the total cost across the full range of model thresholds to identify the global cost minimum.

To propagate predictive uncertainty into the cost estimates, we performed 2,000 bootstrap resamples of the independent validation cohort, evaluating the economic model at the selected operating point (sensitivity 80%) at each iteration, providing 95% confidence intervals for all economic outputs. A one‐at‐a‐time sensitivity analysis was performed across five operational parameters (worsening injury prevalence, model discrimination, annual fixed cost, screening capacity, and cost per screen) to quantify the impact of parameter uncertainty on the economic model.

We emphasize that these parameters serve to demonstrate the modeling framework rather than provide definitive costings. The model provides a transferable decision‐support tool applicable to diverse trial contexts by substituting context‐specific inputs.

### Ethics and funding

Collection and analysis of human samples from the MAPP2 biobank was approved by the London South East Research Ethics Committee (18/LO/0894). Collection and analysis of healthy donor samples obtained through the Centre for Inflammation Research Blood Donor Register, University of Edinburgh, was approved by the Edinburgh Medicine and Veterinary Medicine Research Ethics Committee (21‐EMREC‐041). Evaluation samples were provided from pre‐intervention serum obtained in the ongoing MAIL Trial (Macrophages Therapy for Acute Liver Injury), a clinical trial approved by North East (York) Research Ethics Committee (reference 23/NE/0019), NHS Lothian Research and Development department and the MHRA. This study was funded by the Centre for Precision Cell Therapy for the Liver (PRaCTicaL), a Chief Scientist Office funded research center (PMAS/21/07), and via the Medical Research Council funded MAIL Trial (MR/T044802/1). Funders had no role in writing the manuscript or the decision to submit for publication. No payment was received for writing this article. Authors were not precluded from accessing data in the study and accept responsibility to submit for publication. For the purpose of open access, the author has applied a Creative Commons Attribution (CC BY) license to any Author Accepted Manuscript version arising from this submission.

## RESULTS

### Baseline characteristics

Three DILI phenotypes were defined by peak ALT concentration observed during admission: no DILI (ALT remained less than the upper limit of normal), sub‐hepatotoxic DILI (in which a rise in ALT was evident, but remained sub‐hepatotoxic), and hepatotoxic DILI (peak ALT ≥1,000). This upper threshold was chosen as it is a widely accepted indicator of significant, acute hepatocellular injury (**Table** **1**).^15^

**Table 1:** Baseline characteristics of patients in the discovery and evaluation cohorts

Patient phenotype	Healthy control	No DILI (Peak ALT <50)	Sub‐hepatotoxic DILI (Peak ALT 50–999)	Hepatotoxic DILI (Peak ALT ≥1,000)	Evaluation cohort
Number of patients	13	15	13	20	10
Total number of samples	13	45	28	74	34
Sample time range since final APAP ingestion (h)	N/A	0.5–50.1	2.0–82.0	12.7–154.6	2.8–119.5
Peak ALT during admission (U/L) (median, IQR)	N/A	20 (17–23)	122 (112–195)	3,856 (2473–5,645)	5,155 (2255–7,044)
Median ALT of samples (U/L) (median (IQR))	32 (30–40)	16 (13–20)	146 (105–337)	2,255 (1288–3,653)	3,799 (1118–5,648)

ALT, alanine aminotransferase (U/L); APAP, acetaminophen; DILI, Drug‐induced liver injury; IQR, interquartile range.

### Characterization of novel biomarker profiles in APAP DILI

**Figure 2:**
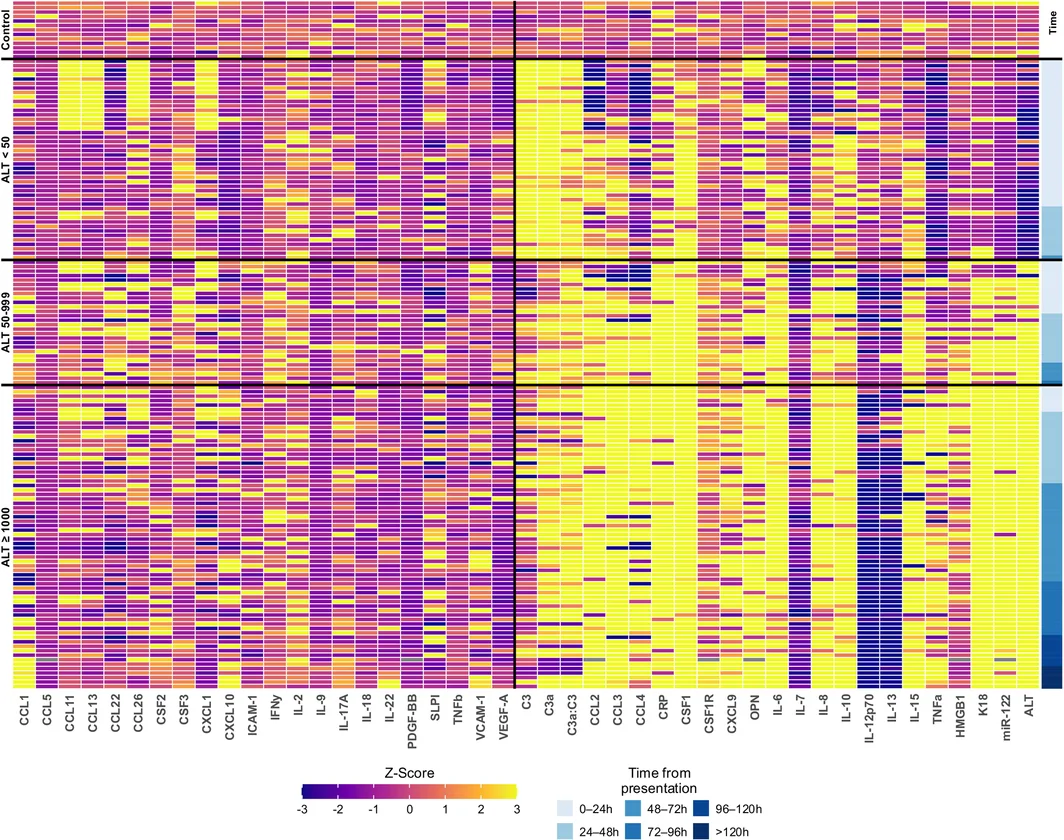
Novel biomarker expression following paracetamol ingestion, stratified by injury phenotype. Heatmap of novel biomarker values. Values are expressed in *n* = 154 samples as median‐adjusted deviation Z‐scores derived from control values. Biomarker phenotype (far left) describes the peak ALT value reached by patients within that phenotype group. Within each phenotype, samples are ordered according to the time since last reported APAP ingestion. Blue bars on the right of the figure indicate 24 hr periods from last APAP ingestion within each group. To highlight different patterns of response, biomarkers are grouped based on the magnitude of change: those with largely stable or inconsistent change in concentrations versus those with distinct evidence of change in APAP DILI. *n* = 6 ALT ≥1,000 phenotype samples are not shown as there was no reported time of last ingestion.

We studied biomarkers measuring hepatocellular injury, cell signaling, organ function, and metabolic state. Exploratory heatmap analysis revealed multiple biomarkers with dynamic changes across APAP DILI phenotypes and time‐course (**Figure** **2**).

Hepatocyte injury markers (keratin‐18, microRNA‐122) paralleled ALT behavior. Complement C3 and C3a showed complex patterns: elevated in overdose without DILI but depleted early in DILI. Several cytokines/chemokines (CCL2, CCL3, CCL4, IL‐6, IL‐8, IL‐10) elevated proportionally to severity; IL‐12p70 and IL‐13 decreased. CSF1 demonstrated compelling changes across phenotypes, potentially reflecting macrophage recruitment (**Figure** **2**).

### Biomarkers distinguish clinical phenotypes of injury severity

We examined whether biomarkers differentiated clinical phenotypes (no ALT rise, sub‐hepatotoxic, hepatotoxic). All samples from a patient were grouped by peak ALT phenotype regardless of individual timepoint values to identify biomarkers reflecting overall trajectory rather than contemporaneous ALT.

**Figure 3:**
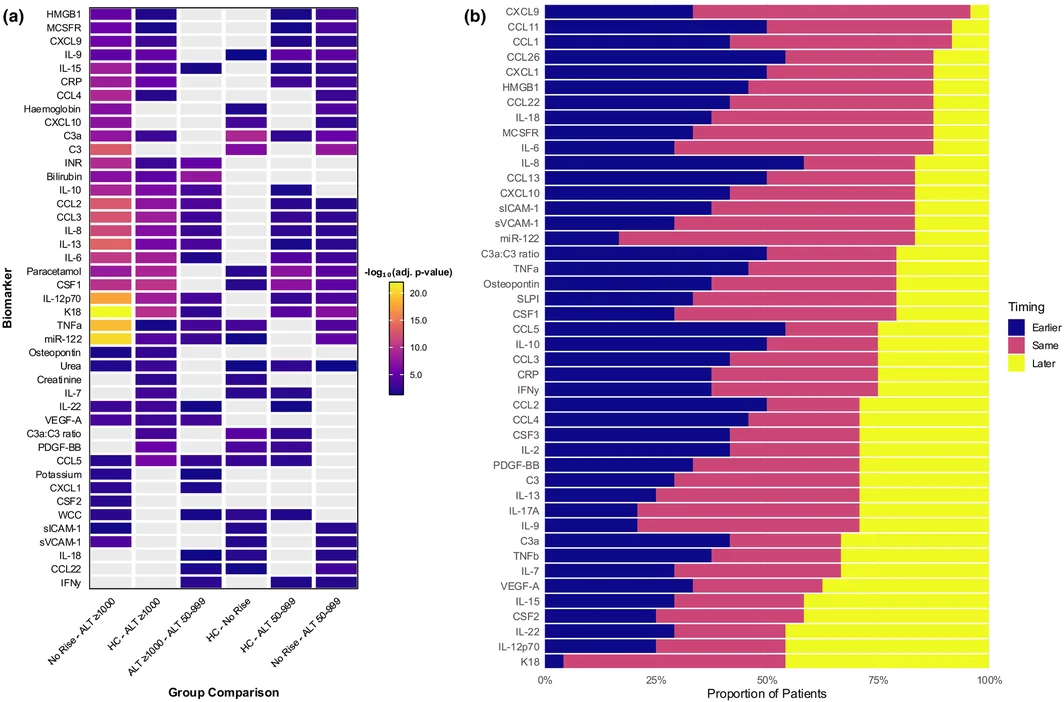
Phenotypic and kinetic differences in biomarkers. (**a**) Biomarkers with a statistically significant difference between phenotype groups obtained by Kruskal–Wallis (*P* < 0.05). Results of *post hoc* comparison tests with adjusted *P* < 0.05 shown in color. Results are ordered by hierarchical clustering. (**b**) Stacked bar chart showing the relative timing of biomarker decline compared to ALT among patients with an identifiable ALT peak (n = 24). Bars represent the proportion of samples in which each biomarker fell earlier than ALT (“wins”), later than ALT (“losses”), or in the same sampling interval (“equal”). Biomarkers are ordered by ascending number of losses. As samples were collected during routine clinical care, time intervals are coarse; simultaneous decline is pragmatically classified as “equal” despite the potential for small timing differences.

Eleven biomarkers were non‐significant by Kruskal–Wallis; remaining biomarkers underwent pairwise comparisons (**Figure** **3** **a**). Inflammation markers (IL‐6, IL‐10), cell death markers (K18, miR‐122), and liver function markers (INR, bilirubin) robustly distinguished hepatotoxic phenotype. Many discriminated all three phenotypes while showing no difference between healthy controls and non‐DILI patients, suggesting utility for severity stratification.

C3a, C3a:C3 ratio, CCL5, and PDGF‐B differed between all APAP‐exposed phenotypes and controls regardless of peak ALT, potentially indicating subclinical injury or APAP exposure effects. CRP, CXCL9, HMGB1, and MCSFR distinguished injury (ALT rise) from uninjured patients but not between sub‐hepatotoxic and hepatotoxic injury, possibly reflecting binary injury transition rather than severity tracking.

Together, these findings demonstrated that many biomarkers change with injury phenotype, and that these relationships may reflect distinct biological processes underpinning the onset, progression, and severity of APAP‐induced liver injury (**Figure** **3**).

### Biomarker time‐course profiles differ from ALT in acute liver injury

We assessed whether biomarkers decline earlier than ALT during recovery, potentially enabling earlier identification of improvement. Analysis included 79 samples from 24 patients with peak ALT ≥50 IU/L and prior samples. We determined whether each biomarker fell before, after, or simultaneously with ALT (**Figure** **3** **b**).

No individual biomarker consistently fell earlier or later than ALT, suggesting that multidimensional biomarker models are required to predict the behavior of ALT. Weighted win ratios suggested IL‐8, CCL26, and CCL11 fell earliest most frequently.

Results demonstrate that biomarker kinetics differ from ALT. While no single biomarker consistently declined earlier across patients, kinetic variability suggests that cohort refinement or biomarker combinations could provide earlier trajectory insights.

### Relationships between biomarkers inform redundancy and complementarity

We next examined the relationships between biomarkers to assess potential redundancy or complementarity. Strongly correlated biomarkers may reflect a shared biological origin, such as the concurrent release of ALT, K18, and miR‐122 during hepatocyte necrosis, and consequently may provide limited added value when combined in a predictive model. (12)^11^ In contrast, biomarkers with weak or no correlation may describe distinct biological pathways.

**Figure 4:**
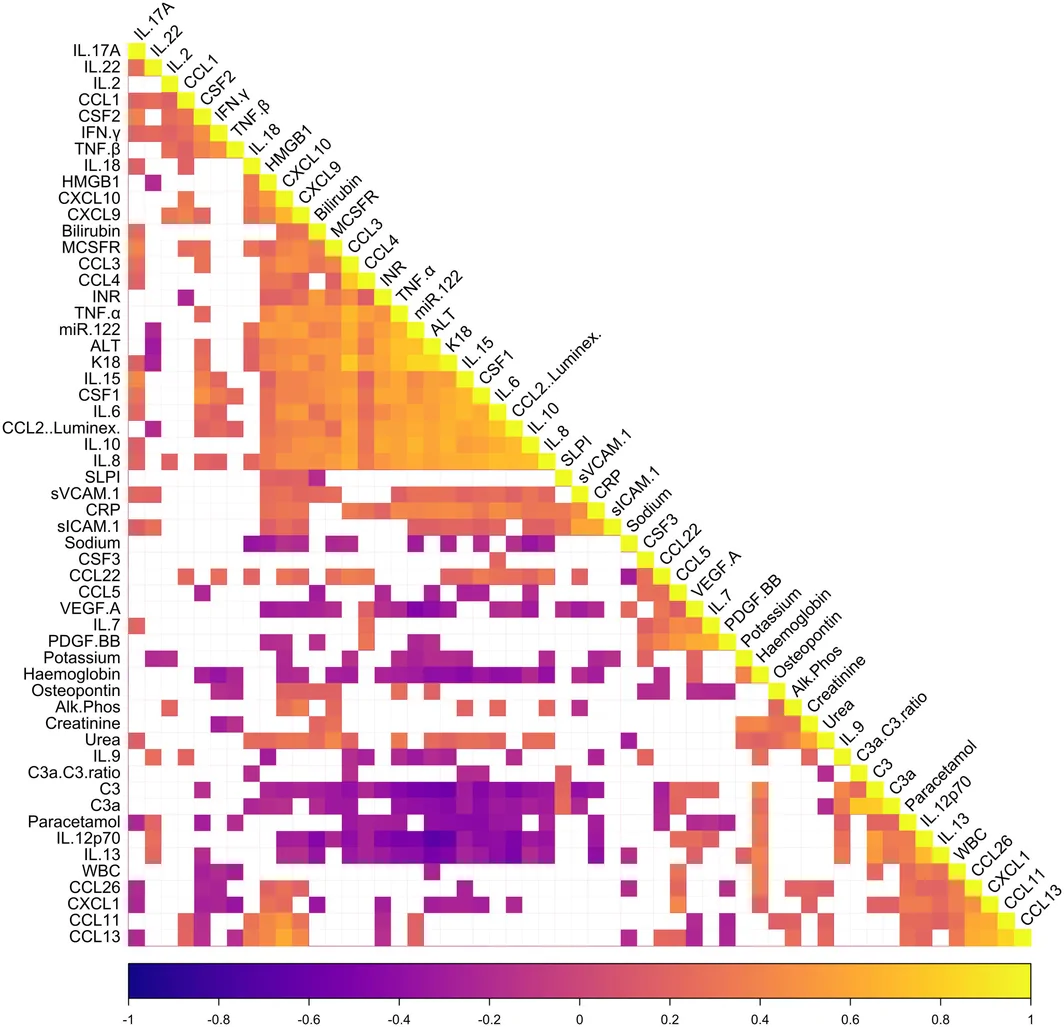
Correlogram of all analyzed biomarkers. Biomarkers are clustered by correlation; only significant correlations (*P* < 0.05) are shown, and are color coded by the strength of correlation as assessed by Spearman's rank correlation.

Pairwise correlations in patients with peak ALT ≥50 (**Figure** **4**) showed clustering among hepatocyte injury markers but weak correlations elsewhere, including many with non‐significant ALT correlation.

This lack of strong correlation, even among biomarkers associated with injury phenotype, suggests magnitude alone does not explain behavior. Temporal profiles and pathophysiological origins may allow certain biomarkers to provide complementary information, particularly for trajectory prediction rather than current state assessment.

These findings support the use of multidimensional biomarker models, where uncorrelated or partially correlated biomarkers can be leveraged to improve prediction of organ injury trajectory. While routine clinical biomarkers are essential for identifying and monitoring liver injury, the added value of novel biomarkers in contextualizing why and how this injury is developing is essential for improved predictive power.

### Dimensionality reduction captures phenotypic clustering, but not injury trajectory

We examined whether dimensionality reduction (PCA, UMAP) considering all biomarkers could distinguish phenotypes and predict trajectory (**Supporting Information** **S3**).

Dimensionality reduction successfully separated patients by injury severity (**Figure** **S3‐3AB**) but failed to identify trajectory, even within the hepatotoxic group (**Figure** **S3‐3CD**). While biomarker concentrations reflect injury state, signals predicting future course are more subtle than unsupervised clustering can resolve.

This prompted supervised modeling. Direct comparison between ALT‐rising and ALT‐falling samples confirmed multiple biomarkers differed significantly between trajectories (**Tables** **S3, S4**), motivating predictive model development.

### Multidimensional biomarker models predict injury trajectory in APAP DILI

#### Concept exploration

Given unsupervised method failure, we developed a supervised machine learning approach to resolve injury trajectory. Using samples following hepatotoxicity development (ALT >1,000 IU/L), we trained a kernel naïve Bayes classifier to predict whether the subsequent ALT measurement would be rising or falling.

ALT alone poorly predicted trajectory (AUC 0.625, 95% CI: 0.441–0.810). Individual novel biomarkers performed better, with IL‐10 and CCL5 achieving AUC 0.783 (**Table** **S4‐1**). Testing pairwise combinations with low inter‐correlation substantially improved performance: 68 pairs outperformed the best single biomarker (**Table** **S4‐1**), supporting multidimensional model development.

#### Model discovery

To optimize model stability for independent evaluation, we performed sensitivity analysis examining the relationship between maximum permitted Spearman correlation (ρ) and evaluation performance. This analysis revealed that a threshold of ρ ≤ 0.5 best optimized the balance between information gain and redundancy (**Supporting Information** **S5**).

**Figure 5:**
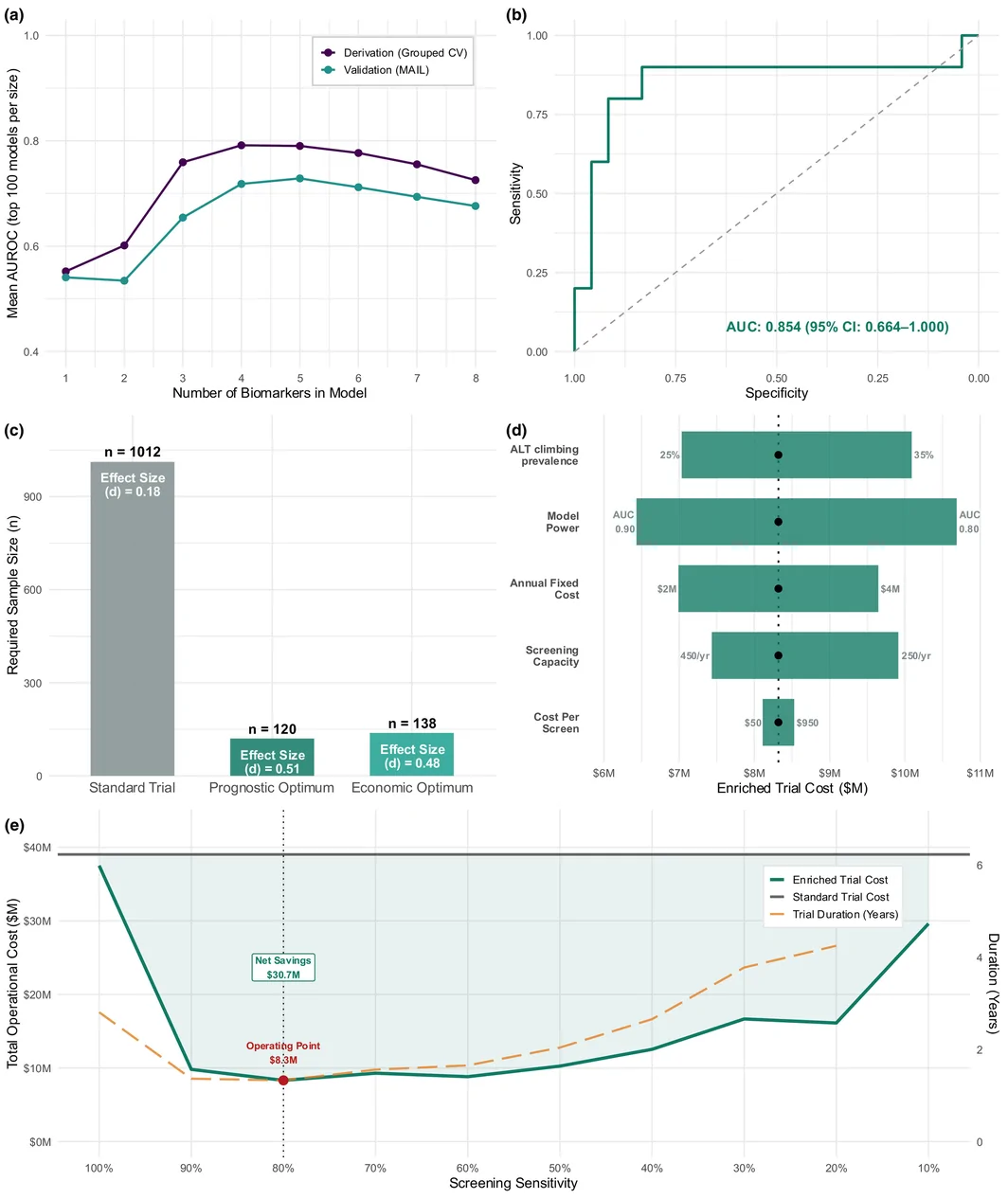
Model development and evaluation, with decision thresholds selected through cost‐minimization modeling simulating a phase 3 clinical trial. (**a**) Sensitivity analysis: Mean AUROC of the top 100 discovery models at derivation and evaluation, against model size, identifying similar trends to the unrestricted search space in Supplemental Information S4. (**b**) receiver operating characteristic (ROC) curve for the selected 4‐biomarker model (K18, MCSFR, Sodium, WBC) at derivation, and subsequent evaluation on the MAIL trial cohort (n = 34), achieving AUC = 0.854 (95% CI: 0.664–1.000). (**c**) Statistical impact: Prognostic enrichment removes noise from spontaneous recovery. At the maximum possible likelihood ratio, the observable effect size (**d**) is restored from d = 0.18 (diluted) to d = 0.51 (enriched). At the economic optimum identified in (**e**), some of this enrichment is traded for economic efficiency. (**d**) Sensitivity analysis: Tornado plot displaying the impact of varying key biological and operational parameters on the projected trial cost. The central dashed line represents the model values (arithmetic mean of plotted limits). Drug effect size (**d**) is excluded: it scales both enriched and standard trial costs equally, preserving the enrichment ratio regardless of the assumed effect magnitude. (**e**) Economic modeling: Continuous analysis of Total Trial Cost and Duration as a function of screening stringency. The model incorporates hypothetical costs described in Methods. The shaded region represents the value added compared to a non‐enriched trial baseline of $39.0 M. The optimal design point yields a projected net saving of $30.7 M while maintaining a feasible recruitment timeline.

With this optimized threshold, we derived models using an exhaustive search for combinations of 1–8 biomarkers. Analysis of the 100 top‐performing models at each size revealed that mean performance peaked at four biomarkers before declining at higher complexities, consistent with the limited pool of non‐collinear biomarkers available in this panel (**Figure** **5** **a**). With a wider pool of mechanistically distinct biomarkers in the derivation set, the derivation performance plateau would be expected to occur at higher model complexities (**Supporting Information** **S4**). The top‐performing ρ ≤ 0.5 biomarker combination at derivation was a 4 biomarker model (MCSFR, WBC, Sodium, K18) with AUC = 0.868.

While a large number of models were evaluated relative to the modest sample size, only the leading model at derivation was applied to the MAIL cohort for independent evaluation.

#### Model evaluation in an independent cohort

We evaluated the performance of the selected four‐biomarker candidate signature (MCSFR, WBC, Sodium, and K18) in the independent MAIL trial cohort (*n* = 10 rising, *n* = 24 falling). This cohort featured only samples obtained pre‐intervention, representing the real‐world population a trialist would face at the point of recruitment, and not confounded by treatment administered in the trial. The population distributions of the discovery and evaluation cohort biomarker peak values, durations of admission, and rates of acute liver failure are provided in **Supporting Information** **S6**.

In this evaluative framework, the candidate model achieved an AUC of 0.854 (95% CI: 0.664–1.000) (**Figure** **5** **b**). Crucially for trial design simulation, the model demonstrated a Positive Likelihood Ratio of 14.4; this metric is independent of prevalence and indicates a robust ability to rule in worsening trajectory. Mechanistically, this signature integrates potential biomarkers of macrophage activation (MCSFR), systemic immune response (WBC), hepatorenal/comorbidity status (sodium), and hepatocellular necrosis (K18), providing a focused assessment of the patient's pathophysiological state.

### Feasibility modeling of economic impact of prognostic enrichment

We distinguished between two decision thresholds: first, the model‐intrinsic optimum maximizing prognostic certainty (sensitivity 60.0%, specificity 95.8%, PPV 85.7%, LR+ 14.4, Enrichment Ratio 2.9x), and second, the economically optimal threshold identified by time‐dependent economic modeling as the global cost minimum. While the former demonstrates the model's discriminatory ceiling, the latter represents the pragmatic deployment strategy that balances statistical efficiency against recruitment feasibility.

To quantify the translational value of this prognostic enrichment strategy, we performed an illustrative economic analysis modeling the trade‐off between statistical efficiency and operational feasibility (**Figure** **5** **c–e**). In a standard trial design (assuming a baseline worsening prevalence 29.4% as seen in the MAIL Trial samples), the dilution of the efficacy signal by spontaneous recoverers necessitates a sample size of 1,012 patients to achieve 80% power (assuming a target effect size of Cohen's d = 0.60). This baseline design results in a projected total trial cost of $39.0 million.

While stricter enrichment (lower sensitivity, higher specificity) theoretically reduces the required sample size, our time‐dependent cost modeling revealed that the impact of other criteria disproportionately increases the consequent costs. Optimizing for this, the model identified a global economic optimum at a sensitivity of 80.0% (95% CI: 66.7–92.9%) and specificity 91.7 (95% CI 72.7–100.0%). At this optimal design point, the enrichment strategy increases the positive predictive value (PPV) for worsening injury from 29.4% to 80% (95% CI: 53.8–100.0%).

Crucially, this threshold maintains a high eligibility yield, resulting in a Number Needed to Screen (NNS) of 3.4 (95% CI: 2.3–4.5). In this context, the NNS represents the operational burden of the enrichment strategy: for every 3.4 patients screened, one eligible patient with a high probability of worsening injury is identified and randomized. This strategy reduces the required sample size to 138 patients while maintaining rapid recruitment velocity. As modeled in **Figure** **5** **e**, a tool with this performance profile projects a total trial cost of $8.3 M (95% CI: $5.8–$15.8 M), delivering a net saving of $30.7 M (95% CI: $23.2–33.2 M) compared to the standard design while ensuring the trial remains operationally deliverable.

## DISCUSSION

Predicting the trajectory of acute organ injury remains a fundamental barrier to the successful clinical translation of novel therapeutics. A high rate of spontaneous recovery creates substantial noise that dilutes efficacy signals in clinical trials. This necessitates large sample sizes to demonstrate a treatment effect, leading to the “valley of death” where biologically plausible therapies fail due to trial design limitations rather than lack of efficacy.^12^, ^16^

In this proof‐of‐concept study, we demonstrate that multidimensional biomarker models have the potential to resolve this heterogeneity. By integrating four mechanistically distinct biomarkers (MCSFR, WBC, Sodium, and K18), we developed a prognostic enrichment tool capable of predicting liver injury trajectory with high accuracy in an evaluation cohort (AUC 0.858), offering a framework to de‐risk future trials.

Alanine aminotransferase (ALT) is the gold standard for diagnosing liver injury, but our data confirm it is a poor tool for predicting future trajectory (AUC 0.625). This limitation arises because ALT reflects current hepatocellular necrosis but fails to capture the broader host response determining recovery or progression. In contrast, a four‐biomarker candidate signature achieved an AUC of 0.854 in an independent evaluation cohort, with a derivation–evaluation gap of only 0.014. The superior performance of this model stems from a principle we term “mechanistic triangulation.” We explicitly optimized for low collinearity (ρ ≤ 0.5) during feature selection. Rather than combining biomarkers that measure the same process, our model integrates independent biological signals: K18 (cell death), WBC (systemic immune response), MCSFR (macrophage activation and regenerative signaling), and sodium (osmoregulatory and hepatorenal status).^17^, ^18^ While unsupervised methods could stratify static severity, they failed to predict dynamic trajectory. It was only through supervised modeling of these distinct axes of pathophysiology that we could resolve the direction of travel for individual patients.

In the discovery cohort, the four selected biomarkers each showed biologically coherent associations with injury trajectory. Two markers of active inflammation and hepatocellular damage (white blood cell count and keratin‐18) were elevated in patients with worsening injury, consistent with ongoing inflammatory infiltration and hepatocyte death, respectively. Conversely, two markers associated with recovery were elevated in patients with resolving injury: MCSFR, possibly reflecting activation of restorative macrophage populations, and sodium, whose normalization from the hyponatremia characteristic of severe hepatic dysfunction may indicate improving liver function.

Operationalizing this model for real‐time trial recruitment requires bridging the gap between bench‐top immunoassays and bedside decision‐making.^19^ While our testing utilized standard ELISA and Luminex platforms, the Number Needed to Screen of 3.4 suggests high throughput. A Companion Diagnostic to support the clinical application of any marketed product would represent a critical next step.^20^ Such a tool would allow trialists to screen, stratify, and randomize patients within the tight therapeutic windows mandated by acute liver injury.

The selection of the optimal decision threshold revealed a critical “time–cost paradox” inherent to prognostic enrichment. While maximizing specificity would theoretically create the purest cohort, doing so disproportionately increases the screening burden. Our economic modeling demonstrated that as sensitivity drops below 40%, the number of screens required to identify eligible patients would rise exponentially. This imposes a duration tax: the savings achieved by treating fewer patients are eventually outweighed by the fixed operational costs of keeping clinical sites open for the extended recruitment period. Consequently, the optimal design point identified a Number Needed to Screen of 3.4, representing an economic equilibrium where the marginal cost of additional screening is balanced against the marginal savings in trial size and duration, favoring a high‐sensitivity strategy over a restrictive rule‐in approach.

Regulatory guidance from the FDA supports enrichment strategies to improve trial efficiency, but acute organ injury trials have previously focused on identifying disease phenotypes likely to respond, rather than identifying disease trajectory to decrease statistical noise.^18^ Our economic simulation illustrates the impact of implementing this type of model as a screening tool. In a hypothetical Phase 3 efficacy trial, prognostic enrichment raised the baseline probability of worsening injury from 29% to 80%, increasing the signal‐to‐noise ratio, conceivably supporting a reduction in sample size from 1,012 to 138 patients.

In addition, this study provides a generalizable methodological framework for biomarker model discovery in complex diseases. By filtering for low collinearity prior to model assembly, we maximized information gain while minimizing redundancy.

## LIMITATIONS

These findings should be interpreted in the context of study limitations. While the model was validated in an independent cohort (MAIL trial), the sample size of the evaluation set consisted of 34 samples. We consider this an exploratory evaluation of the candidate signature; larger‐scale prospective validation would be required before regulatory qualification as a companion diagnostic for a licensed therapeutic.

Our discovery and evaluation cohorts consisted of survivors, who are only partially characterized according to the Model for End‐Stage Liver Disease (MELD). While this signature may theoretically differ in patients with rapid progression to fulminant failure, this survivor‐heavy phenotype represents the precise target population for Phase 2/3 efficacy trials: patients with significant injury where the window for therapeutic salvage remains open. The absence of patients with established, irreversible necrosis reflects limitations of the sample cohorts available, rather than a deliberate modeling choice, but the result is a model optimized for a cohort in which interventions aim to alter clinical trajectory, rather than salvage end‐stage organ failure. Patients with fulminant hepatic failure represent a distinct clinical trajectory requiring separate prognostic modeling, and extending the current framework to include mortality prediction represents an important direction for future work.

The analysis utilized serum samples collected during routine care, resulting in coarse time intervals between sampling and introducing sample volume constraints, which left us unable to quantify all possible desirable biomarkers, such as alpha‐fetoprotein, mitochondrial specific biomarkers, paracetamol adducts, and carbamoyl phosphate synthetase 1. We also note that our economic modeling assumes a specific effect size and cost structure; actual savings will vary by trial design and clinical setting.

Finally, our model was developed and evaluated exclusively in the context of dose‐dependent paracetamol hepatotoxicity. The pathophysiology of idiosyncratic drug‐induced liver injury differs substantially in mechanism, time‐course, and immune involvement; consequently, the biomarker panel and decision thresholds derived here should not be assumed to generalize to other forms of DILI without dedicated validation in those populations.

## CONCLUSION

We describe a multidimensional biomarker model that predicts the trajectory of acetaminophen‐induced liver injury with greater accuracy than current standard‐of‐care markers. By distinguishing patients destined for spontaneous recovery from those with worsening injury, this tool enables prognostic enrichment with the potential to reduce the sample size and cost of clinical trials. This “mechanistic triangulation” approach offers a tangible solution to a translational bottleneck in acute liver failure, providing a pathway to accelerate the development of new therapies.

## FUNDING

Chief Scientist Office, Scotland (PMAS/21/07); UK Medical Research Council (MR/T044802/1).

## CONFLICT OF INTEREST

JWD: Patent on a new single‐biomarker liver toxicity point‐of‐care test. SJF: co‐founder of Resolution Therapeutics, which is developing a macrophage cell therapy product to treat patients at risk of liver decompensation. CH: Elsevier honorarium for educational article authorship, advisor to Royal College of Emergency Medicine and Joint Royal Colleges Ambulance Liaison Committee on Toxicology. AMK: Consultant to Resolution Therapeutics. All other authors declared no competing interests for this work.

## AUTHOR CONTRIBUTIONS

C.H., A.M.K., S.J.F., and J.W.D. wrote the manuscript; C.H. designed the research; C.H., A.M.K., K.M.S., R.A., L.B., M.E.C., and T.Y.M. performed the research; C.H. and A.M.K. analyzed the data.

## Supporting information


Table S1.

Data S1.

## Data Availability

The authors declare that all data supporting the findings of this study are available within the paper and its supplemental information. Requests for further data and analytical code may be addressed to the corresponding author.
